# Gold nanoparticles combined baker’s yeast as a successful approach for breast cancer treatment

**DOI:** 10.1186/s43141-023-00481-1

**Published:** 2023-03-06

**Authors:** Amany Elwakkad, Amina A. Gamal el Din, Hisham A. Saleh, Noha E. Ibrahim, Mohamed A. Hebishy, Hagar H. Mourad, Mahitab I. El-Kassaby, Howida Sayed Abou-Seif, Ghada M. Elqattan

**Affiliations:** 1 Medical Physiology Department, Medical Research and Clinical Studies Institute, National Research Centre, 33 El-Bohouth St. (El-Tahrir St. Former), Giza, 12622 Dokki Egypt; 2 Pathology Department, Medical Research and Clinical Studies Institute, National Research Centre, 33 El-Bohouth St. (El-Tahrir St. Former), Giza, 12622 Dokki Egypt; 3 Electron Microscope and Thin Films Department, Physics Research Institute, National Research Centre, 33 El-Bohouth St. (El-Tahrir St. Former), Giza, 12622 Dokki Egypt; 4 Microbial Biotechnology Department, Biotechnology Research Institute, National Research Centre, 33 El-Bohouth St. (El-Tahrir St. Former), Giza, 12622 Dokki Egypt

**Keywords:** Breast cancer, Gold nanospheres, Apoptosis, Baker’s yeast

## Abstract

**Background:**

*Saccharomyces cerevisiae* (*S. cerevisiae*) has been demonstrated in vitro to sensitize several breast cancer cell lines and to be a safe, non-toxic drug with anti-skin cancer action in mice. Furthermore, plasmonic photothermal treatment using gold nanorods has been authorized as a novel method for in vitro and in vivo cancer therapy.

**Results:**

When compared to tumor-free rats, the treatment with *S. cerevisiae* conjugated to gold nanospheres (GNSs) lowered Bcl-2 levels while increasing FasL, Bax, cytochrome c, and caspases 8, 9, and 3 levels. Histopathological results showed changes reflecting the ability of nanogold conjugated heat-killed yeast to induce apoptosis is greater than heat-killed yeast alone as the nanogold conjugated with heat-killed yeast showed no tumor, no hyperplasia, no granulation tissue formation, no ulceration, and no suppuration. Nanogold conjugated with heat-killed yeast-treated breast cancer group displayed normal levels of ALT and AST, indicating relatively healthy hepatic cells.

**Conclusion:**

Our results proved that nanogold conjugated heat-killed yeast can initiate apoptosis and can be used as a safe non-invasive method for breast cancer treatment more effectively than the yeast alone. This, in turn, gives us new insight and a future hope for the first time that breast cancer can be treated by non-invasive, simple, safe, and naturally originated method and achieves a hopeful treatment and a novel method for in vivo cancer therapy.

## Background

Breast cancer (BC) is one of the most prevalent types of cancer in women throughout the world [1]. The incidence of BC has been growing at a 0.3% yearly pace in recent years, with 2.3 million new cases expected in 2020 and a considerable rise in mortality from 1990 to 2015 [2]. It is caused by estrogen/progesterone receptor mutations [1]. It symbolizes a malignant tumor in which breast cells proliferate uncontrollably and drive out normal cells [3]. It begins in the interior layer cells (epithelium) of the glandular tissue’s ducts (85%) or lobules (15%). For start, the malignant progression is limited to the duct or lobule (in situ), where it generates no symptoms and has little potential for dissemination (metastasis). These in situ (stage 0) tumors may develop and infiltrate neighboring breast tissue (invasive breast cancer), and then spread to nearby lymph nodes (regional metastasis), or other organs in the body (distant metastasis). Breast cancer causes death in women owing to extensive metastases [4]. Although modern therapies can eradicate the initial tumor, many patients still die from metastasis and tumor relapse. This is due to a modest population of unique cells in cancer tissue that are identical to normal stem cells and play a significant role in tumor creation and progression. Self-renewal, multi-differentiation potential, high proliferation capability, and high oncogenicity suggest that they may be the cause of cancer carcinogenesis, recurrence, and metastasis [5].

7,12-dimethylbenz(a)anthracene (DMBA) is a hydrocarbon that has been used to develop tumors in animals as a carcinogenic and immunosuppressive agent. It boosts the formation of prostaglandin E2 (PGE2), which raises the prevalence of breast cancers, as well as other carcinogenic features such as disruption of cellular oxidant-antioxidant balance and mutation [6]. It possesses strong carcinogenic and immunosuppressive properties and is commonly utilized as a model of polycyclic aromatic hydrocarbons (PAHs)-induced breast cancer [7]. DMBA causes cancer by either starting or encouraging mutations in cancer-causing genes [8]. Furthermore, DMBA causes oxidative stress by generating reactive oxygen species (ROS) and peroxides as a result of DMBA enzymatic activation [9], and this oxidative stress is important in carcinogenesis [10] aside from liver and renal damage caused by the lipid peroxidation process [11]. Because of its carcinogenic impact, DMBA is utilized to develop breast cancer because it reduces apoptosis by upregulating BCL2 and downregulating apoptotic genes (Bax, caspase-3) [12].

To date, there have been relatively few therapeutic choices for treating severe stages of the disease. Cancer metastasizes, lowering survival rates, and sadly, therapy is primarily palliative. As a result, finding apoptotic agents for metastatic breast cancer (MBC) cells with minimal side effects is critical [13]. Science practically all anticancer drugs have significant side effects on normal tissues and organs, tumor recurrence and the spread of malignant cells are highly prevalent following regular cancer therapy [14]. As seen by the significant death rate of women with this malignancy, conventional therapy is significantly less than optimum. Traditional treatment options, including surgery, radiation, and chemotherapy, are not as successful as predicted, raising worries about limited bioavailability, low cellular absorption, growing resistance, and severe effects [1].

Previously, baker's yeast, *Saccharomyces cerevisiae* (*S. cerevisiae*), was shown to cause apoptosis in numerous tumor cell lines, including breast [15, 16], colon [17], and tongue [18, 19], but had no impact on normal cells [20]. *S. cerevisiae* is a powerful anti-cancer apoptotic agent. Cancer cells undergo apoptosis after *S. cerevisiae* phagocytosis; it can also have anticancer effects in nude mice bearing human breast cancer [18, 19] and in Swiss albino mice bearing Ehrlich carcinoma [13]. In both murine and human breast cancer cells, yeast generates anticancer effects. It might be utilized as an adjuvant for chemotherapy, which could have therapeutic implications for breast cancer treatment [21].

Elwakkad et al. (2018) demonstrated that baker’s yeast (*S. cerevisiae*) is a safe, non-toxic drug that displays anti-skin cancer action in mice via intrinsic and extrinsic apoptotic mechanisms, suggesting its potential utility in the treatment of cancer in humans after clinical trials [22].

Nanobiotechnology has ushered in a new era of nanomedicines and is assisting in the resolution of various medical issues [23]. It is essential in the treatment of chronic illnesses such as cancer, diabetes, AIDS, and TB [24].

A nanomedicine-based approach to breast cancer treatment is a potential new option. Nanomedicine as a platform for studying innovative therapeutic applications and advanced intelligent healthcare management systems is advancing rapidly in the modern era [1]. The most promising aspect of cancer nanotechnology is the ability to construct nanovehicles with numerous compounds that, because of their tiny size, may enter tumors with great selectivity and, as a result, with much fewer side effects [3]. Because of their unique optical, magnetic, electrical, and structural features, gold nanoparticles (GNPs) have been shown to have a prospective utility in the field of biomedical research [25]. The ability of medicines, antibodies, and other compounds to be readily loaded on GNPs surfaces makes GNPs uses in medicine possible [26]. In addition, they are non-toxic, and the body can eliminate them via the kidneys. Many researchers have been conducted to investigate the use of GNPs in cancer treatment [27, 28]. NG has been shown to have tremendous potential for individuals suffering from metastases following breast cancer [29].

Recent research found that mice injected with NG (1–9 nm) had smaller tumors and a greater survival rate than untreated control mice [30]. It has been demonstrated that when GNPs enter tumor tissue, they aggregate and destroy the targeted tumor cells [31].

Up until now, there is no available literature on the application of *S. cerevisiae* conjugated to gold nanospheres (*S. cerevisiae*-GNSs) for either in vitro or in vivo treatment of breast cancer and for the application of *S. cerevisiae*-GNSs in cancer therapy it is necessary to know the systemic toxicity associated with their use. Therefore, the goal of this work is to (1) examine the biotherapeutic capabilities of *S. cerevisiae*-GNSs therapy, against breast cancer in rats. (2) Clarify the molecular processes behind *S. cerevisiae*-GNSs impact. (3) Investigate the indirect effect of *S. cerevisiae*-GNSs on breast cancer cell development via immunological regulation and antioxidant capacity. (4) Study any possible toxic effects of *S. cerevisiae*-GNSs on the blood profile and the liver and kidney functions in rats.

## Materials and methods

### Experimental animals

From four to 6-week-old female Wister albino rats, about 75–100 gm each, were obtained from the animal house of the National Research Centre, Dokki, Giza, Egypt. Rats were housed in plastic cages, accommodated for 1 week before experimentation and permitted to acclimate in standard conditions (under 12-h light/dark cycles).

The animals were given free access to distilled water and commercialized food all over the experiment. The environmental conditions were standardized concerning temperature, humidity, and light. All animal procedures were conducted as stated by the guidelines established by the National Health and Medical Research Council and were approved by the Institutional Animal Ethics Committee of the National Research Centre, Giza, Egypt, registration number 19–204. The animals were divided into 4 groups, 25 rats/group, as follows:

The first group, normal control rats without tumors served as a negative control. The second group bearing tumor was untreated and served as a positive control. The third group was intratumorally (IT) injected with 100 µl/tumor *S. cerevisiae* at a concentration of 10^9 ^cells/ml yeast twice\week for 16 weeks and the fourth group was IT injected by *S. cerevisiae* conjugated to GNSs twice\week for 16 weeks.

The rats in groups 1 and 2 were sacrificed at the end of the induction period, and the rats in groups 3 and 4 were sacrificed at the end of 16 weeks post-treatment.

### Tumor induction

After 1 week of accommodation, animals were injected with 7, 12–dimethylbenz[α] anthracene (DMBA) (purchased from Sigma Chemicals Company, USA). Single dose of 50 mg/kg b. w. in 2 ml of corn oil subcutaneously injected in the mammary gland of healthy rats (average weight 120–130 gm). Then rats were allowed to develop tumors for 120 days.

### Preparation of gold nanoparticles conjugated to yeast

Gold nanoparticles solution was prepared by the reaction carried out through the following steps:

HAuCl4 with a volume of 800 μL of 1 g/50 ml, 80 ml of distilled water is added to 8 ml of yeast solution with gentle shaking and a white-purple solution was obtained. Ice-cold NaBH4, 80 μL of 0.0378 g/10 ml, is injected at once into the above mixture. The color of the mixture is instantly turned from white-purple to reddish brown.

Ascorbic acid, 16 μL of 0.138/10 ml, is added to this aqueous mixture, which results in changing the growth solution from white purple to colorless. Finally, the color changes slowly within 40–55 min to reddish purple.

The ultraviolet–visible absorption spectra (UV–Vis.) of GNPs solution were measured using JASCO corp., V-570 spectrophotometer over a spectral range 180–1200 nm with an accuracy of ± 0.1 nm [32, 33]. All spectra were recorded at room temperature. The maximum absorption spectrum for regular, spherical-shaped colloidal Au was between 500 and 530 nm because the resonant excitation of plasmons is affected by the surface of nanoparticles. Transmission electron microscope (TEM) study includes transmission electron micrographs, and electron diffraction (ED) studies were carried out for GNSs by using JEOL JEM-1230 electron Microscope, with maximum resolving power 0.2 nm, energy 40–120 kV, maximum magnification power × 600,000 [34, 35]. From the TEM results, it is very important to make sure that most of the nanoparticles made have sphere shapes and not rods or other shapes.

### Preparation of yeast (*S. cerevisiae*)

Active dry baker’s yeast, *S. cerevisiae*, was prepared by suspending yeast (1 gm/100 ml) in phosphate-buffered saline (PBS). The suspensions were heat-killed via incubation for 1 h at 90 °C and subsequently washed twice with PBS and re-suspended in PBS.

Quantification was done by using a hemocytometer. Yeast cell suspensions were adjusted to 10^9^ cells/ml and rats received IT injection of 100 μL yeast [22].

### Histopathological preparation

At the end of the experiment, animals were sacrificed. Mammary gland tissue, liver, and kidneys were dissected and extracted from the sacrificed animals. Organ tissues were fixed in 10% buffered formalin for 48 h, processed through ascending grades of alcohol, cleared in xylene, and prepared into paraffin blocks. Serial Sects. 5 microns thick each were prepared from each block and stained with hematoxylin and eosin (H&E) for routine histopathologic study [36].

The sections were examined using an Olympus CX41 research microscope. To avoid any bias, observations of the histopathologic examination were interpreted by an experienced observer by blinding the sample identity.

Slide tissue microphotography was done using CCD digital camera (Olympus SC100) attached to the microscope. Digital photomicrographic sections were taken at various magnifications.

### Genetic preparation

#### Quantitative real-time polymerase chain reaction (qRT-PCR) for analysis of tumor protein P53 (p53), tumor necrosis factor-alpha (TNF-α), and B cell lymphoma 2 (Bcl-2) mRNA expression.

Total RNA was isolated from 30–45 mg of breast tissues of rats by using RNeasy mini Kit (Qiagen; USA; Cat No. 74104), by following the manufacturer’s instructions, and stored at − 20 °C. The RNA concentration and purity were determined by Nanodrop Spectrophotometer absorption (Thermo Scientific, USA) at 260 nm [37].

Gene expression of Bcl2, TNF-α, and P53 has carried out in the presence of β-actin housekeeping gene [Sequences of both forward and reverse primers are described in Table 1. QuantiTecht SYBR Green Master Mix (Qiagen; USA; Cat No. 204243) was used for one-step RT-PCR quantification. The reaction was performed by Stratagene Mx3000 P QPCR instrument (Agilent Technologies, Santa Clara, CA, USA). Briefly, in a 50-μl reaction volume, 4 μl of extracted RNA were added to 25 μl of 2 × SYBR Green Master Mix, 0.5 μl of quantities RT mix, and 200 ng of each primer. The Thermal cycle of RT-PCR software was as follows: 50 °C for 30 min, 95 °C for 15 min, and 94 °C for 15 s., with an annealing temperature of 57 °C for 20 s and final extension at 72 °C for 15 s for 40 cycles. The relative expression of the current amplified genes was obtained using the comparative thermal cycle (2^−ΔΔCT^) method [38].

**Table 1:** Primers sequence used in RT-PCR analysis

Gene	Forward	Reverse
Bcl2	5-GACAGAAGATCATGCCGTCC-3	5-GGTACCAATGGCACTTCAAG-3
TNF-α	5′-CCAGACCCTCACACTCAGATCA-3′	5′-TCCGCTTGGTGGTTTGCTA-3′
P53	5′-CTGTCATCTTCTGTCCCTTC-3′	5′ TGGAATCAACCCACAGCTGCA3′
β-actin	5-CTTTGATGTCACGCACGATTTC-3	5-GGGCCGCTCTAGGCACCAA-3

### Serum and organs collection

Blood samples were collected from the retro-orbital plexus of rats under diethyl ether anesthesia in clean tubes. The 1st time was at the end of the induction period for the rats bearing tumors untreated beside the normal control rats without tumors; and the 2nd time was at the end of the experiment for the treated groups for the examination of the different pathological and biochemical assays.

All tubes were left to clot at room temperature and then centrifuged at 4000 r.p.m for 15 min. The separated serum was collected and kept at – 80 °C until analysis to examine different biochemical assays. Organs (mammary gland, liver, and kidneys) were collected in 10% buffered formalin and sent for histopathological examinations. Parts of breast tissue were preserved at – 80^ º^C for genetic analysis and preparation of tissue homogenates.

### Preparation of tissue homogenates

Tissue homogenates were prepared as follows: rats bearing tumor, with and without treatment, were sacrificed; samples of breast tissue (5–10 mm^3^) were cut; weights of the tissues were recorded; and breast tissues were rinsed with ice-cold PBS (0.01 M, pH = 7.4), weighed, minced and then homogenized in PBS (9 mL PBS per gram tissue) with a glass homogenizer on ice. The homogenates were centrifuged at 3000 r.p.m for 10 min. and supernatants were removed and frozen at − 80 °C.

### Experimental design

Female Wister albino rats under different treatment conditions were examined for the following: (1) biochemical analysis for the apoptotic pathway. (2) Histopathological analysis. (3) Genetic analysis. (4) Toxicity that includes (A) animal behavior, (B) animal survival, (C) histopathological analysis of internal organs, and (D) biochemical analysis.

The obtained data were statistically analyzed using GraphPad Prism. The values were presented as the mean ± SD [39].

## Results

### Characterization of gold nanospheres (GNSs)

**Fig. 1:**
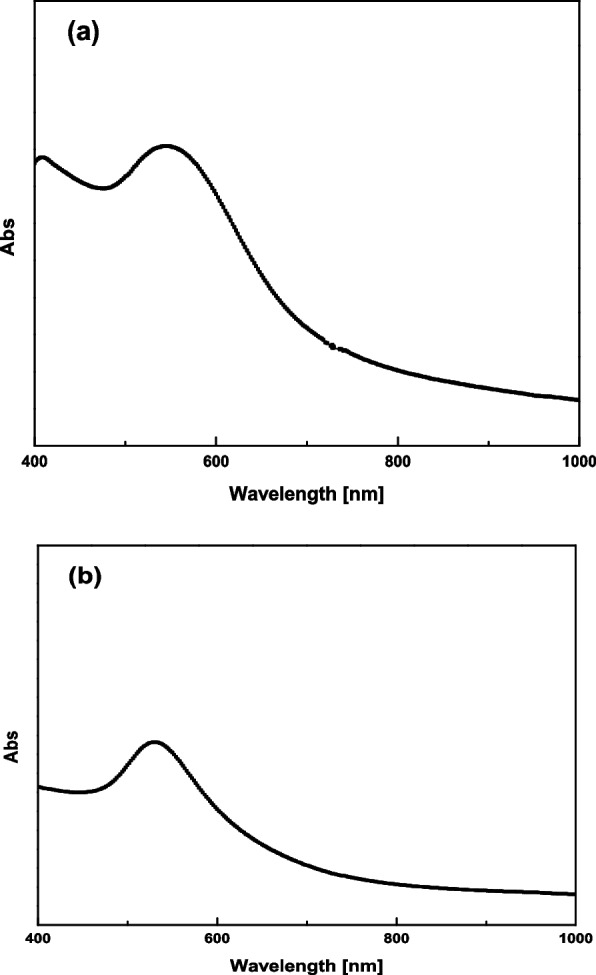
**a** Absorption spectra of aqueous GNS solution after 45 min. **b** GNS solution after a long time

**Fig. 2:**
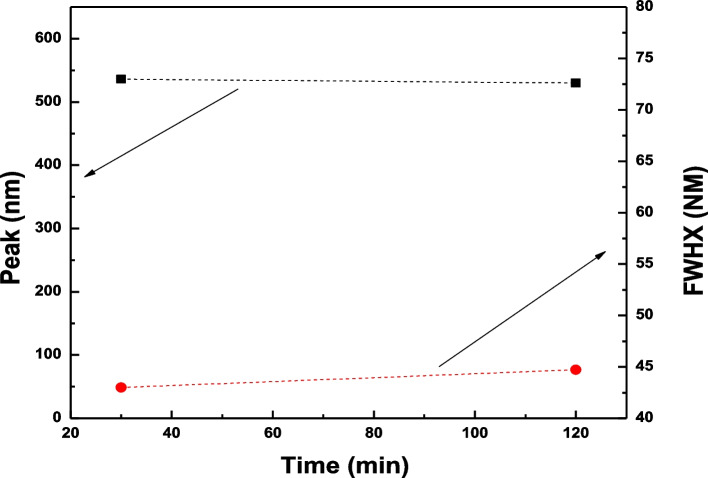
The main extinction peak and the FWHM (full width at half-maximum) as a function of time

Figure 1 depicts the optical absorption spectra of the GNSs solution in the visible near-IR region. The spectrum exhibits one distinct peak at 530 nm. Au nanospheres exhibited one surface plasmon peak, and there is a consistent localized surface plasmon resonance (LSPR) shift toward the light in the red spectrum from nanospheres. The optical absorption spectrum of the GNSs solution did not change its properties after a long time. This increases the stability of the solution and prevents unexpected changes in its properties. From Fig. 2, the main extinction peak is the same after a long time, and the FWHM (full width at half-maximum) of the distinct peak increased with time; this means that the gold sphere particle preparation is on the scale of nanoparticles.

**Fig. 3:**
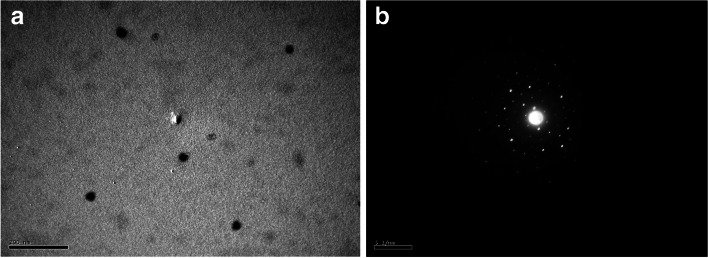
**a** TEM images of GNSs and **b** electron diffraction

TEM image of the prepared GNSs is presented in Fig. 3. It was noticed that some non-agglomerated GNSs were formed. From Fig. 3a, these GNSs were, to a large extent, homogenous in shape and size. The obtained nanosphere averaged 20–25 nm. Figure 3b shows that the electron diffraction of the prepared GNSs is crystalline.

**Fig.4:**
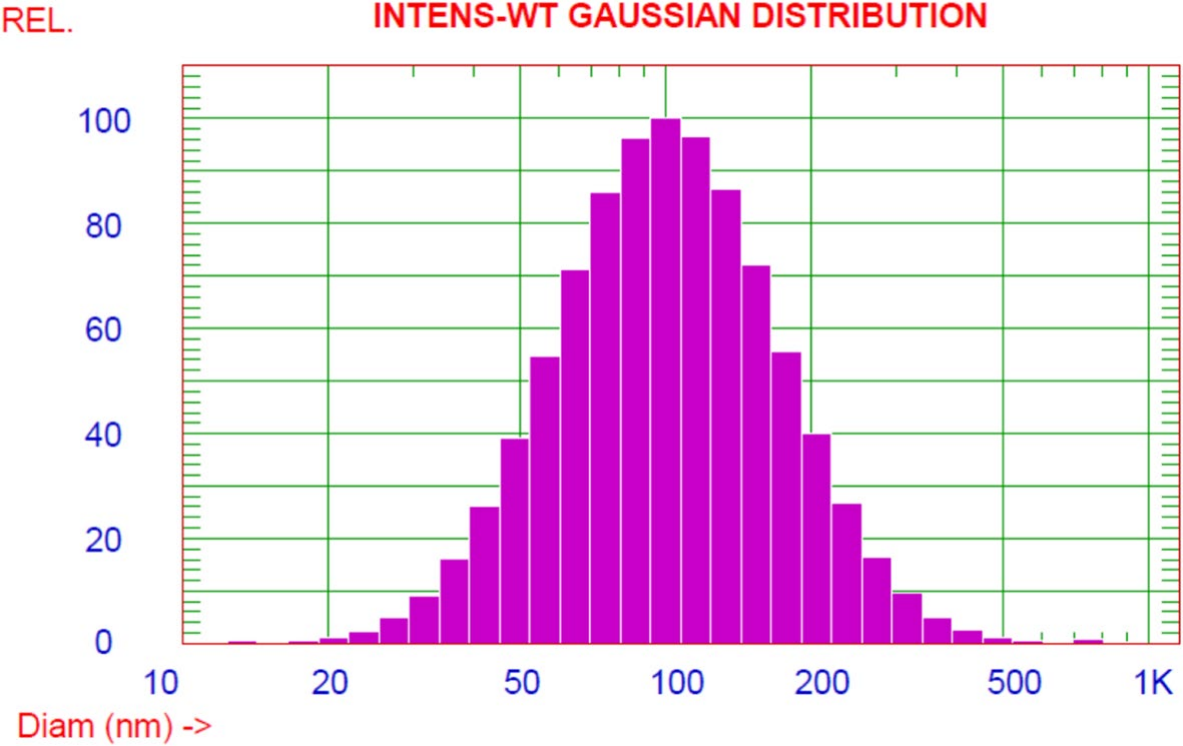
Zeta potential and particle size distribution of nanogold

The size distribution of the as-prepared samples was examined using the dynamic light scattering (DLS) technique by Nicomp Nano DLS/ZLS Systems, as shown in Fig. 4. The average diameter of *S. cerevisiae*-GNS was found to be 115 nm, which is greater than that estimated from TEM images. DLS calculated the hydrodynamic size, which is the size of the nanoparticle plus the liquid layer around it [40].

The zeta potential was used to investigate the stability of colloidal *S. cerevisiae*-GNS. The negative zeta potential of *S. cerevisiae*-GNS was − 29.52 mV. These values are within the permitted range for nanoparticle dispersions. The negative zeta potential values demonstrated by gold nanoparticles might be ascribed to the putative capping of biomolecules present in *S. cerevisiae* [41].

### Biochemical analysis of the apoptotic pathway

#### Analysis of the apoptotic regulators

The expression of pro-apoptotic proteins FasL and Bax, as well as anti-apoptotic protein Bcl-2, was studied in rats under various treatment conditions; tumor-free rats served as the negative control. Rats with untreated tumors served as positive controls, the third group was intratumorally (IT) injected with 100 μl/tumor *S. cerevisiae* at a concentration of 10^9^ cells/ml, and the fourth group was IT injected with *S. cerevisiae*-GNSs.

### Bcl-2 (ng/ml)

Bcl-2 expression was considerably higher in tumor homogenates than in normal control tissue (*P* ≤ 0.01) (Table 2). However, as compared to rats carrying tumors alone, treatment with *S. cerevisiae* at 10^9^ produced a reduction in Bcl-2 expression (*P* ≤ 0.01). In comparison to tumor tissue homogenate, a reduction in Bcl-2 level was seen after 100 µl of *S. cerevisiae*-GNSs treatment (*P* ≤ 0.01). The comparison of Bcl-2 levels in rats treated with *S. cerevisiae* alone and those treated with *S. cerevisiae*-GNSs revealed that Bcl-2 expression was much lower in the *S. cerevisiae*-GNSs treated animals, with a highly significant effect (*P* ≤ 0.01) in comparison to those treated with yeast alone.

**Table 2:** Effect of intratumoral injection with 10^9^ cells/ml of *S. cerevisiae* and *S. cerevisiae*-GNSs on Bcl-2 concentration in the negative, positive, and treatment groups

Parameter	Bcl-2 (ng/ml)			
Groups	Mean ± S.E	Significance to normal	Significance to tumor	Significance to yeast group
Negative control (normal)	1.35 ± 0.07	–––––-	##	+
Positive control (tumor)	4.73 ± 0.33	**	–––––-	+ +
*S. cerevisiae* 10^9^ (yeast group)	1.04 ± 0.01	*	##	–––––-
*S. cerevisiae*–GNSs	0.55 ± 0.06	**	##	+ +

Significance relative to normal: *P ≤ 0.05 and **P ≤ 0.01; significance relative to tumor: ##*P* ≤ 0.01; significance relative to yeast: + *P* ≤ 0.05 and +  + *P* ≤ 0.01

### FasL (ng/ml)

Table 3 reveals that the concentration of FasL in tumor homogenate in rats with tumor alone is significantly lower than in normal control homogenate (*P* ≤ 0.01). In comparison to the positive control, FasL expression increased considerably after yeast 10^9^ therapy (*P* ≤ 0.01). Similarly, FasL values in treated rats with *S. cerevisiae*-GNSs increased significantly (*P* ≤ 0.01) when compared to the positive control group. When compared to the yeast group, FasL expression increased significantly (*P* ≤ 0.05) in *S. cerevisiae*-GNSs treated-animals.

**Table 3:** Effect of intratumoral injection with 10^9^ cells/ml of *S. cerevisiae* and *S. cerevisiae*-GNSs on FasL concentration in the negative, positive, and treatment groups

Parameter	FasL (ng/ml)			
Groups	Mean ± S.E	Significance to normal	Significance to tumor	Significance to yeast group
Negative control (normal)	19.33 ± 1.45	–––––-	##	+
Positive control (tumor)	6.40 ± 0.83	**	–––––-	+ +
*S. cerevisiae* 10^9^ (yeast group)	24.00 ± 2.08	*	##	–––––-
*S. cerevisiae*–GNSs	29.00 ± 2.08	*	##	+

Significance relative to normal: *P ≤ 0.05 and **P ≤ 0.01; significance relative to tumor: ##*P* ≤ 0.01; significance relative to yeast: + *P* ≤ 0.05 and +  + *P* ≤ 0.01

### Bax (ng/ml)

Table 4 demonstrated that the levels of Bax in the homogenates of the positive control group decreased slightly (*p* ≤ 0.05) from the normal control. When compared to untreated rats, treatment with either *S. cerevisiae* at a concentration of 10^9^ cells/ml or *S. cerevisiae*-GNSs generated a very highly significant increase in Bax level (*P* ≤ 0.001). Although this increase was more visible in the animals treated with *S. cerevisiae*-GNSs. Treatment with *S. cerevisiae*-GNSs, in contrast, resulted in an increase in Bax concentration (*P* ≤ 0.01) if compared to the yeast group.

**Table 4:** Effect of intratumoral injection with 10^9^ cells/ml of *S. cerevisiae* and *S. cerevisiae*-GNSs on Bax concentration in the negative, positive, and treatment groups

Parameter	Bax (ng/ml)			
Groups	Mean ± S.E	Significance to normal	Significance to tumor	Significance to yeast group
Negative control (normal)	250.00 ± 5.77	–––––-	#	+ +
Positive control (tumor)	143.43 ± 12.09	*	–––––-	+ + +
*S. cerevisiae* 10^9^ (yeast group)	370.00 ± 5.77	**	###	–––––-
*S. cerevisiae*–GNSs	546.67 ± 24.04	**	###	+ +

Significance relative to normal: *P ≤ 0.05 and **P ≤ 0.01; significance relative to tumor: #*P* ≤ 0.05 and ###*P* ≤ 0.001; significance relative to yeast: ^++^*P* ≤ 0.01 and ^+++^*P* ≤ 0.001

### Executive of apoptosis

#### Cytochrome c (pg/ml)

Table 5 shows that the levels of cytochrome c in homogenates of the untreated group decreased significantly more than those in the normal rats (*p* ≤ 0.01). A concentration of *S. cerevisiae* (10^9^cells/ml) caused a considerable rise in cytochrome c levels (*p* ≤ 0.01). Treatment with *S. cerevisiae*–GNSs caused a sixfold increase in cytochrome c (*p* ≤ 0.001) and this increase is more noticeable than in the *S. cerevisiae* group (*p* ≤ 0.05).

**Table 5:** Effect of intratumoral injection with 10^9^cells/ml of *S. cerevisiae* and *S. cerevisiae*-GNSs on Cytochrome c concentration in the negative, positive, and treatment groups

Parameter	Cytochrome c (pg/ml)			
Groups	Mean ± S.E	Significance to normal	Significance to tumor	Significance to yeast group
Negative control (normal)	7.68 ± 0.38	–––––-	##	+
Positive control (tumor)	4.73 ± 0.33	**	–––––-	+ +
*S. cerevisiae* 10^9^ (yeast group)	15.75 ± 1.58	*	##	–––––-
*S. cerevisiae*–GNSs	24.13 ± 1.26	**	###	+

Significance relative to normal: *P ≤ 0.05 and**P ≤ 0.01; significance relative to tumor: ##*P* ≤ 0.01 and ###*P* ≤ 0.001; significance relative to yeast: ^+^*P* ≤ 0.05 and ^++^*P* ≤ 0.01

### Quantification of caspases 9, 8, and 3 (ng/ml)

Caspases 9, 8, and 3 concentrations were measured in the positive control, 10^9^cells/ml yeast-treated and yeast conjugated to GNSs groups.

A decrease in the levels of caspases 9, 8, and 3 was noticed in the untreated rats when compared to normal. Rats given 10^9^cells/ml yeast had higher levels of caspases 9, 8, and 3, particularly caspase 9 (*p* ≤ 0.01). Similarly, caspases 9, 8, and 3 levels were considerably raised in the group treated with yeast conjugated to GNSs, with caspases 9 and 8 levels being higher (*p* ≤ 0.01) (Table 6).

**Table 6:** Effect of intratumoral injection with 10^9^cells/ml of *S. cerevisiae* and *S. cerevisiae*-GNSs on caspase 9, 8, and 3 concentrations in the negative, positive, and treatment groups

Parameters	Caspase 9	Caspase 8	Caspase 3
Groups			
Negative control (normal)	29.67 ± 0.33^#,+^	2.73 ± 0.12^##,+^	3.47 ± 0.15^#,N.S^
Positive control (tumor)	15.67 ± 2.03^*,++^	1.70 ± 0.15^**,+^	2.03 ± 0.32^*,+^
*S. cerevisiae* 10^9^ (yeast group)	41.00 ± 2.65^*,##^	3.63 ± 0.19^*,#^	4.23 ± 0.32^n.s,#^
*S. cerevisiae*–GNSs	44.00 ± 1.53^**,##^	3.90 ± 0.06^*,##^	4.47 ± 0.26^**,#^

Significance relative to normal: *P ≤ 0.05, **P ≤ 0.01, and n.s *P* ≥ 0.05; significance relative to tumor: #*P* ≤ 0.05, ##*P* ≤ 0.01; significance relative to yeast: + *P* ≤ 0.05, +  + *P* ≤ 0.01 and N.S *P* ≥ 0.05

### Histopathological analysis

Histopathological changes were examined in tumor-bearing rats, in the treated group with 100 μl/tumor *S. cerevisiae* 10^9^ cells/ml and *S. cerevisiae* conjugated to the GNSs group.

**Fig. 5:**
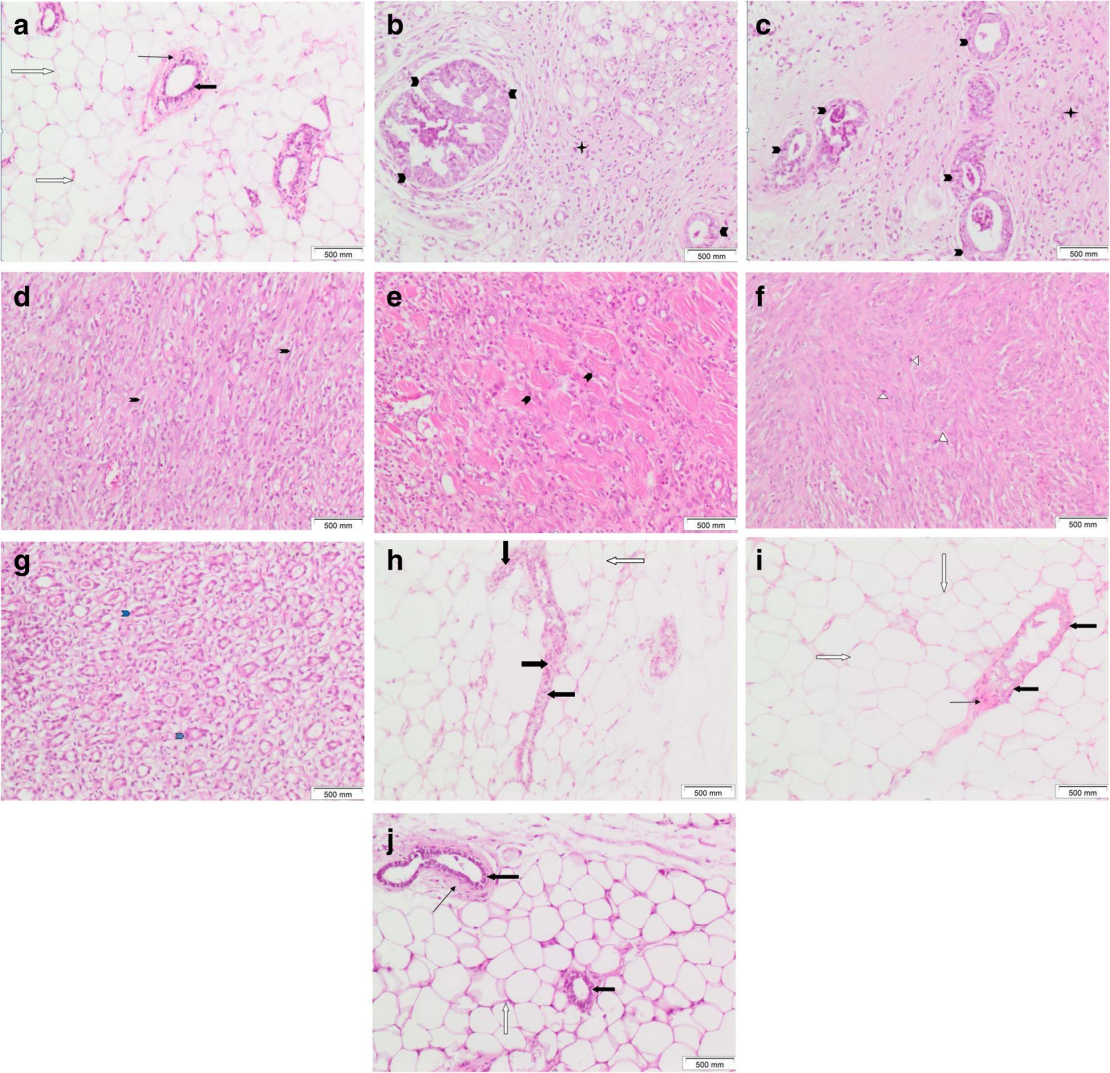
Mammary gland tissue of **A** control female rats shows ordinary benign mammary ducts lined by benign double epithelium and myoepithelium (thick black arrow), surrounded by thin eosinophilic connective tissue (thin black arrow) amidst fat cells (white arrow). **B**–**G** DMBA-injected rats showing **B** DCIS cribriform and clinging patterns (black arrowheads) with mixed inflammatory cell aggregates (a black star) within surrounding tissues. **C** intraductal carcinoma (black arrowheads). **D** Cellular, spindle, and fusiform cell proliferation (black arrowheads). **E** Spindle and fusiform tumorous cell proliferation invading in between muscle bundles (black arrowheads). **F** Cellular, spindle cell tumorous proliferation with several mitoses (white triangles). **G** Tubular tumorous proliferation (blue arrowheads) lined by atypical epithelial cells with intervening fusiform and spindle cells. **H**, **I** Yeast-injected rats showing residual atypical hyperplasia within mammary ductal epithelial lining (thick black arrows). **I** Nanogold carrying yeast injected rats showing mammary ducts with benign lining approximating control (thick black arrows). No tumor (H&E × 100)

**Fig. 6:**
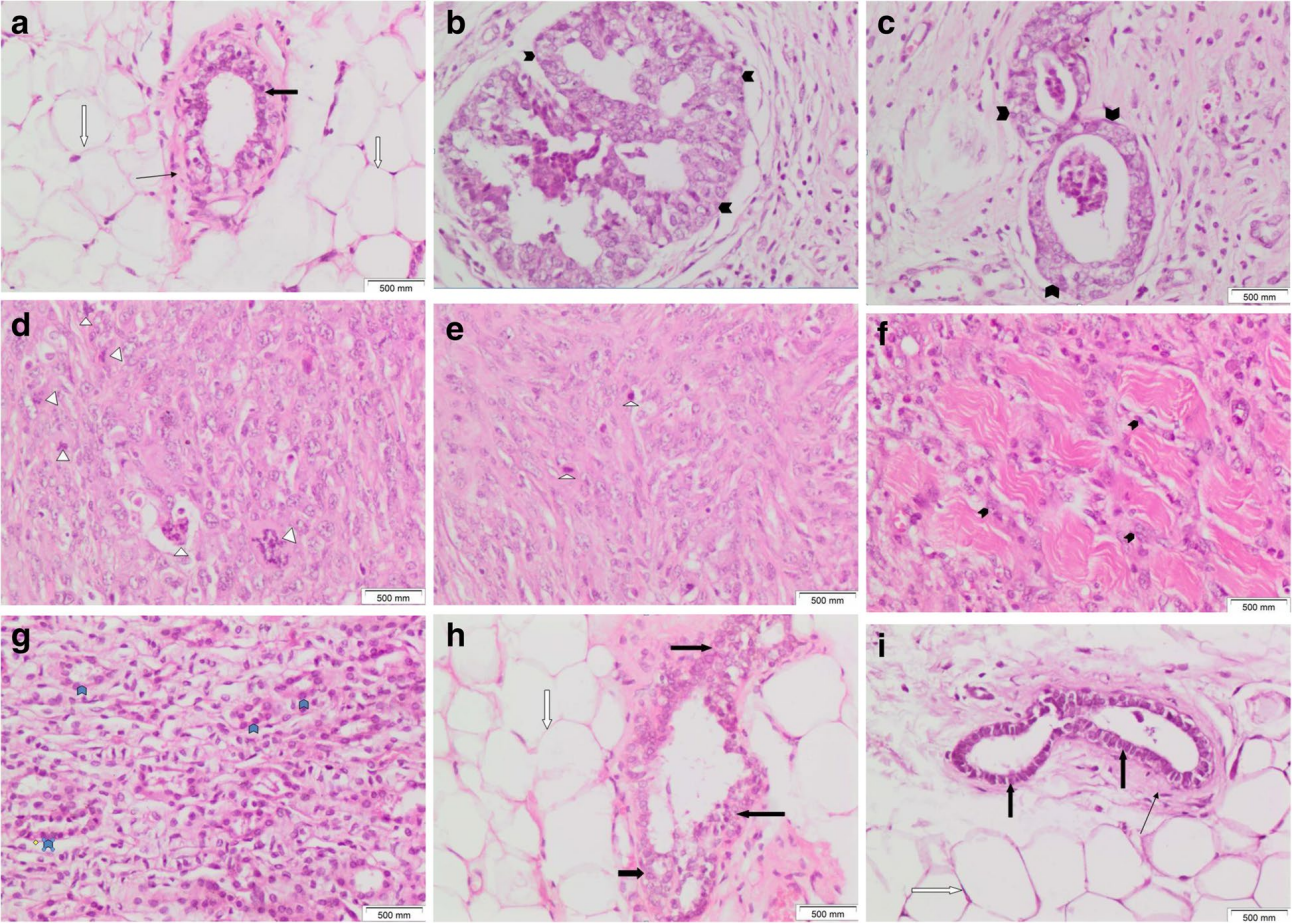
Mammary gland tissue of **A** control female rats showing ordinary benign mammary duct lined by benign double epithelium and myoepithelium (thick black arrow), surrounded by thin eosinophilic connective tissue (thin black arrow) amidst fat cells (white arrow). **B**–**G** DMBA-injected rats showing: **B** DCIS cribriform pattern (black arrowheads). **C** Intraductal carcinoma (black arrowheads). **D**, **E** Pleomorphic, cellular, spindle, and fusiform tumorous cell proliferation with numerous mitoses (white triangles); some being abnormal. **F** Spindle and fusiform tumorous cell proliferation invading in between muscle bundles (black arrowheads). **G** Tubular tumorous proliferation (blue arrowheads) lined by atypical epithelial cells with intervening fusiform and spindle cells. **H** Yeast-injected rats showing residual hyperplasia within mammary ductal epithelial lining (thick black arrows). No tumor. **I** Nanogold carrying yeast-injected rats showing mammary ducts with benign, regular linings approximating control (thick black arrows). No tumor (H&E × 200)

**Fig. 7:**
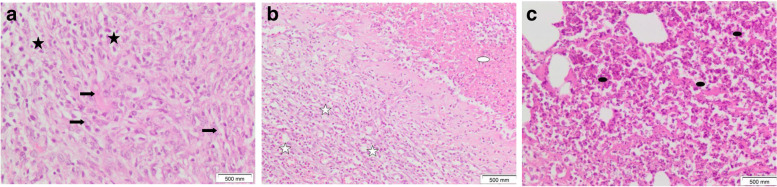
Tissue sections of **A** Mammary gland tumor induced by DMBA injection in rats showed cellular proliferation formed of atypical fusiform and spindle cells (thick black arrows) and inflammatory cells (small black stars). Tissues overlying mammary gland tumor showed **B** wide ulceration and fibrinous exudate formation (white oval shape), exuberant granulation tissue (white scars) with dense inflammatory cellular infiltrate, and **C** dense suppurative inflammation with numerous polymorphs and pus cells (black oval shape) (H&E × 200)

Tissue sections of the control rat mammary gland showed two main components: the epithelium and the stroma. The epithelium consisted of luminal epithelial cells that lined mammary ducts and acini and basal (myoepithelial) cells that formed the outer layer of the gland. Thin eosinophilic connective tissue was seen surrounding the ducts. The stroma consisted of mature adipocytes (Figs. 4A and 5A). Tissue sections of DMBA-injected rat mammary glands showed tumor formation in all rats in the group. Two rats showed DCIS (ductal carcinoma in situ). Ducts were filled with malignant epithelial cells in a prominent cribriform-fenestrated pattern (Figs. 4B and 5B). Other ducts were lined by flat malignant epithelial cells with open lumina (a clinging pattern). Scattered ducts showed Roman bridges while several ducts showed intraluminal papillary structures (Figs. 4C and 5C). The remaining rats showed tumor tissue formed of markedly cellular and pleomorphic, atypical spindle, and fusiform cell proliferation with numerous mitotic figures, several of which were abnormal (Figs. 4D, F, and 5D, E). Atypical cells were observed infiltrating between muscle bundles (Figs. 5E and 6F). Tubular tumor elements were seen lined by atypical epithelial cells with anaplastic fusiform and spindle cells sweeping in between (Figs. 5G and 6G); this picture is consistent with malignant adenomyoepithelioma. Tissues overlying mammary gland tumors induced by DMBA injection in rats showed wide ulceration, secondary infection, fibrinous exudates, dense suppuration, and exuberant granulation tissue formation (Figs. 7B and C).

Mammary tissue sections of rats injected with yeast treatment showed residual atypical ductal hyperplasia with ducts lined by residual atypical hyperplastic epithelial cells (Figs. 5H, I, and 6H). No ulceration, no suppuration, and no granulation tissue formation were found.

No tumor tissue was found in mammary tissue sections of rats injected with nanogold carrying yeast treatment. Ordinary ducts and acini lined by benign double epithelial and myoepithelial cell layers were observed in a picture approximating control (Figs. 5J and 6I). No tumor, no atypia, no hyperplasia, no ulceration, no suppuration, and no granulation tissue formation were observed.

### Genetic results

#### Impact of yeast and yeast conjugated to NGSs on Bcl-2, p53 expression, and the inflammatory cytokine TNF-α

The results deduced that, after applying the ANOVA test, there was a noticeable change in Bcl-2 gene expression fold change within groups (*p* ≤ 0.001). After applying Bonferroni for multiple comparisons, the noticeable change was between normal control and positive control (*p* ≤ 0.001), positive control and yeast (*p* ≤ 0.001), positive control and nanogold conjugated yeast (*p* ≤ 0.001), while there was no noticeable change between yeast and nanogold conjugated yeast (*p* value = 1).

In addition, after applying the ANOVA test, there was a noticeable change in TNF-α gene expression fold change within groups (*p* ≤ 0.001). After applying Bonferroni for multiple comparisons, the significant difference was between the positive control and normal control (*p* ≤ 0.001), positive control and nanogold conjugated yeast (*p* ≤ 0.001), positive control and yeast (*p* ≤ 0.001).

**Fig. 8:**
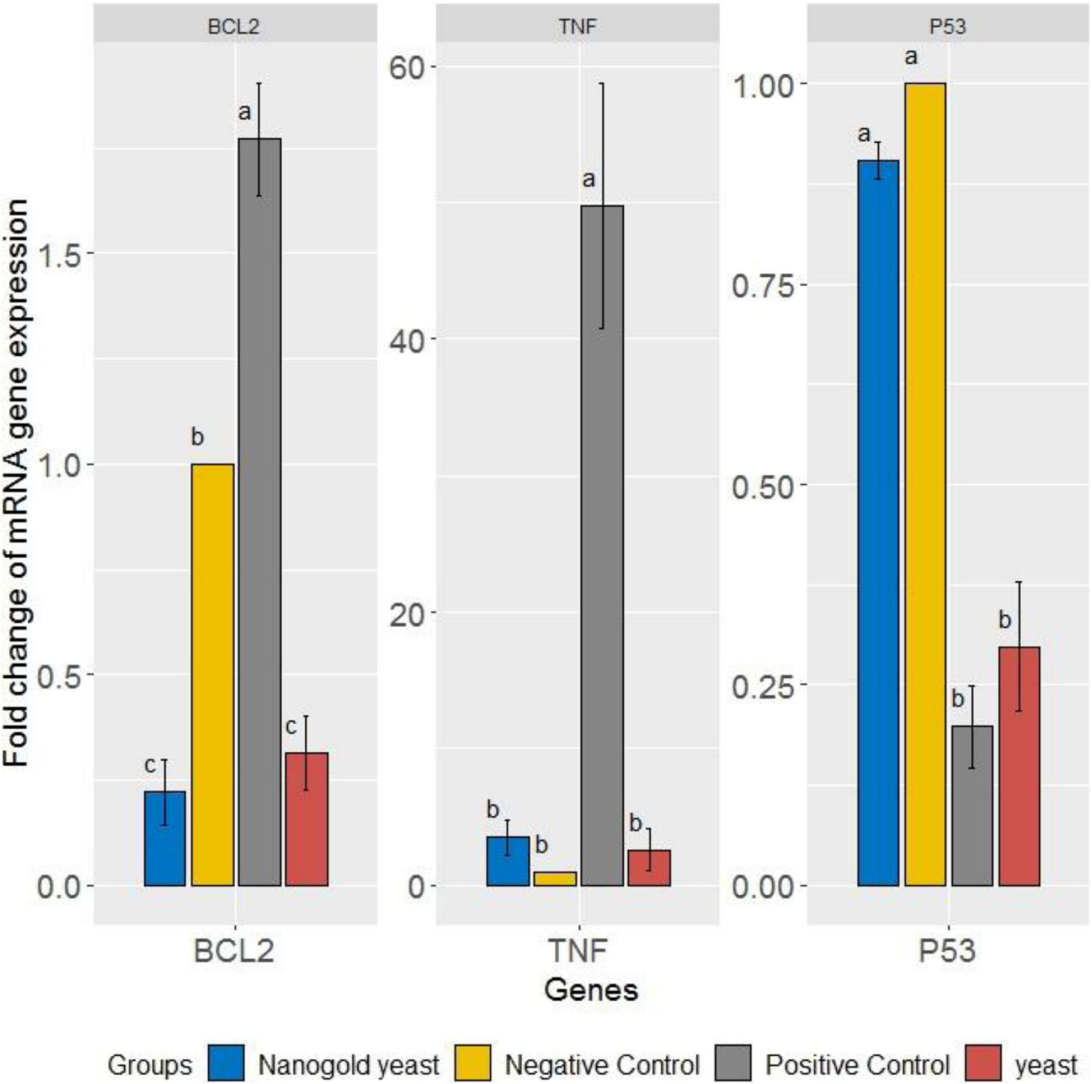
DMBA for the induction of breast cancer in rats caused a significant downregulation in the gene expression of p53 as compared to the control value. Nevertheless, a significant upregulation was apparent in yeast-treated rats and yeast-NGSs. But, DMBA for the induction of breast cancer in rats caused a significant up-regulation in the gene expression of TNF-α and Bcl-2 as compared to the control value and the treated groups (yeast, yeast-NGSs)

In addition, after applying the ANOVA test, there was a noticeable change in p53 gene expression fold change within groups (*p* ≤ 0.001). After applying Bonferroni for multiple comparisons, the significant difference was between normal control and positive control (*p* ≤ 0.001), normal control and yeast (*p* ≤ 0.001), positive control and nanogold-conjugated yeast (*p* ≤ 0.001), nanogold conjugated yeast, and yeast (*p* ≤ 0.001) (Fig 8).

### Toxicity studies

Toxicity tests were performed to verify whether a 10^9^ concentration of yeast and yeast conjugated to GNSs IT treatment is safe to use.

(1) Animal behavior, (2) animal survival, (3) histological investigation of internal organs, and (4) biochemical analysis were all investigated.

### Monitoring animal behavior

The possible harmful side effects of yeast and yeast conjugated with GNSs therapy were observed in animals. Daily examinations revealed that injection of 10^9^ concentration of heat-killed *S. cerevisiae* and yeast conjugated with GNSs gave no adverse side effects. This was evidenced by the fact that life activity patterns, including feeding/drinking of yeast-treated and yeast conjugated to GNSs-treated rats, were recorded for the entire duration of treatment and were found to be normal when compared to normal control rats.

### Monitoring animal survival

Animal survival was monitored on a daily basis. According to the findings of this investigation, all animals that got 10^9^ concentration of yeast, and yeast conjugated to GNSs treatment remained alive over the whole length of the study period. Rats with breast tumors, on the other hand, lived just 2–3 weeks without therapy.

### Histopathological analysis of internal organs

#### Histopathological results of liver

**Fig. 9:**
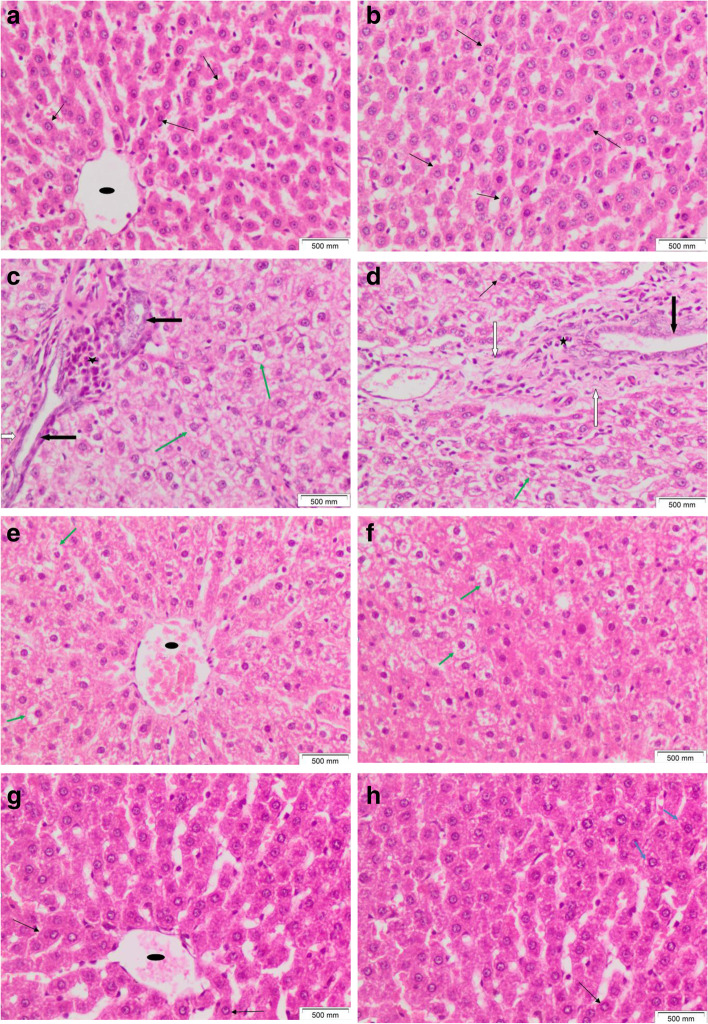
Liver tissue sections of **A**, **B** showing preserved architecture with ordinary hepatocytes (thin black arrows) with small, regular, rounded nuclei disposed in single thick plates, radiating from the central vein (black oval shape). **C**, **D** DMBA-injected rats showed disturbed architecture with prominent hydropic degeneration within hepatocytes (green arrows), proliferating bile ducts (thick black arrows), periductal bands of fibrosis (white arrows), and mixed inflammatory cell aggregates (black star). **E**, **F** DMBA-injected rats treated with injected yeast showed preserved architecture and numerous ordinary liver cells; with minimal residual hydropic degeneration (green arrows) within scattered hepatocytes. **G**, **H** DMBA-injected rats treated with injected nanogold-carrying yeast showed a picture approximating control, with mostly ordinary hepatocytes (thin black arrows) except for minimal microvesicles within a few liver cells (H&E × 200)

Liver tissue sections of the control rat showed preserved architecture. Ordinary hepatocytes with regular, rounded, small nuclei were seen disposed in single-cell thick plates, radiating from the central vein (Fig. 9A, B). Liver tissue sections of DMBA-injected rats showed disrupted architecture. Prominent hydropic degeneration was noticed within hepatocytes. Proliferating bile ducts were seen with periductal bands of fibrosis. Scattered aggregates of mixed inflammatory cells were noticed (Fig. 9C, D).

Liver tissue sections of DMBA-injected rats treated with injected yeast showed preserved architecture. Numerous ordinary hepatocytes were observed admixed with scattered ones showing minimal residual hydropic degeneration. No bile duct proliferation, no fibrosis, and no inflammatory cell aggregates (Fig. 9E, F). Liver tissue sections of DMBA injected rats treated with injected nanogold carrying yeast showed a picture approximating control. Preserved architecture was restored. Mostly ordinary hepatocytes were seen with minimal microvesicles within the cytoplasm of a few ones. No bile duct proliferation, no fibrosis, and no inflammatory cell aggregates (Fig. 9G, H).

### Histopathological results of kidney

**Fig. 10:**
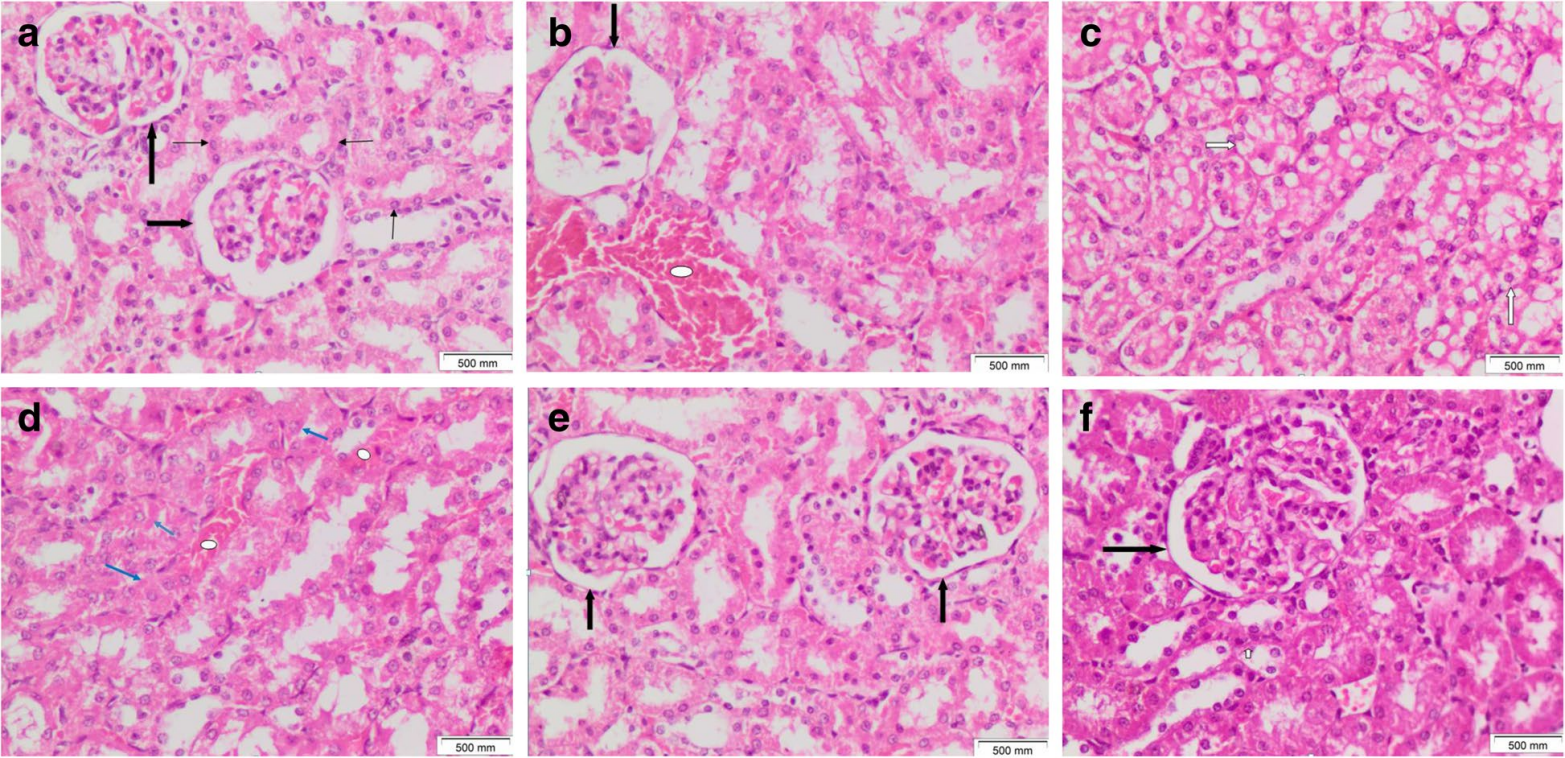
Kidney tissue sections of **A** control rat showing averagely cellular glomeruli (thick black arrows) with surrounding ordinary tubules lined by low cuboidal epithelium (thin black arrows). **B**,** C**, **D** DMBA-injected rats showing congestion within interstitial tissues (white oval shapes), hypocellular glomerulus with reduced mesangium (thick black arrow), tubular lining epithelial vacuolar degeneration (white arrows), and cloudy swelling (blue arrows). **E** Yeast injection treated DMBA-injected rat showing averagely cellular glomeruli (thick black arrows) surrounded by ordinary tubules and **F** nanogold carrying yeast injection treated DMBA-injected rat showing averagely cellular glomerulus (thick black arrow) surrounded by tubules lined by low cuboidal epithelium ((H&E × 200)

Kidney tissue sections of the control rat showed average cellular glomeruli. Surrounding ordinary renal tubules were seen lined by low cuboidal epithelium (Fig. 10A). Kidney tissue sections from DMBA-injected rats showed hypocellular glomeruli with reduced mesangium (Fig. 10B). Surrounding renal tubules showed degeneration within the lining epithelium, including prominent vacuolar degeneration (Fig. 10C) and cloudy swelling (Fig. 10D). Congestion within interstitial tissues was observed as well (Fig. 10B, D).

Kidney tissue sections of yeast injection treated DMBA injected rats showed picture approximating control. Averagely cellular glomeruli were seen surrounded by tubules lined by low cuboidal epithelium (Fig. 10E). Kidney tissue sections of nanogold carrying yeast injection treated DMBA injected rats showed averagely cellular glomeruli surrounded by tubules lined by cuboidal epithelium (Fig. 10F).

### Biochemical analysis

Table 7 shows that tumor-bearing rats had considerably higher levels of AST, ALT, urea, and creatinine than normal rats. However, treating animals with 10^9^ cells/ml yeast and *S. cerevisiae*-GNSs kept these parameters within normal rat ranges.

**Table 7:** Effect of injection intratumorally with 10^9^ cells/ml of *S. cerevisiae* and *S. cerevisiae*-GNSs on AST, ALT, urea, and creatinine concentrations in the negative, positive, and treatment groups

Parameters	AST	ALT	Urea	Creatinine
Groups				
Negative control (normal)	289.0 ± 27.6^n.s,N.S^	246.3 ± 19.8^###,N.S^	50.6 ± 0.4^n.s,N.S^	0.45 ± 0.06^#,N.S^
Positive control (tumor)	411.7 ± 10.9^n.s,+++^	406.2 ± 32.6^***,+^	70.7 ± 4.7^n.s,++^	0.85 ± 0.07^*,+^
*S. cerevisiae* 10^9^ (yeast group)	208.0 ± 3.8^n.s,###^	200.6 ± 34^n.s,#^	46.3 ± 5.2^n.s,##^	0.46 ± 0.02^n.s,#^
*S. cerevisiae*–GNSs	273.3 ± 5.9^n.s,##^	215.4 ± 14.3^n.s,#^	40.1 ± 4.7^n.s,###^	0.31 ± 0.05^n.s,#^

Significance relative to normal: *P ≤ 0.05, ***P ≤ 0.001, and ^n.s^ *P* ≥ 0.05; significance relative to tumor: #*P* ≤ 0.05, ##*P* ≤ 0.01, ###*P* ≤ 0.001; significance relative to yeast: ^+^*P* ≤ 0.05, ^++^*P* ≤ 0.01, ^+++^*P* ≤ 0.001 and ^N.S^*P* ≥ 0.05

Table 8 shows that tumor-bearing animals had considerably greater levels of lipid peroxidation, total antioxidants, and superoxide dismutase than normal rats. Treatment with 10^9^ cells/ml yeast and *S. cerevisiae*-GNSs, on the other hand, kept these parameters within normal rat ranges.

**Table 8:** Effect of injection intratumorally with 10^9^ cells/ml of *S. cerevisiae* and *S. cerevisiae*-GNSs on total antioxidant, SOD, and MDA concentrations in the negative, positive, and treatment groups

Parameters	Total antioxidant	Superoxide dismutase (SOD)	Malondialdehyde (MDA)
Groups			
Negative control (normal)	12.4 ± 0.9^#,N.S^	146.7 ± 5.7^n.s,+^	6.9 ± 1.6^##,N.s^
Positive control (tumor)	8.0 ± 0.3^*,+++^	137.6 ± 3.2^n.s,+^	18.4 ± 1.4^**,+^
*S. cerevisiae* 10^9^ (yeast group)	14.0 ± 0.2^n.s,###^	166.7 ± 2.7^*,#^	6.6 ± 0.9^n.s,#^
*S. cerevisiae*–GNSs	13.3 ± 1.1^n.s,#^	147.3 ± 5.3^n.s,n.s^	9.5 ± 1.4^n.s,#^

Significance relative to normal: *P ≤ 0.05, **P ≤ 0.01, and ^n.s^*P* ≥ 0.05; significance relative to tumor: #*P* ≤ 0.05, ##*P* ≤ 0.01, and ###*P* ≤ 0.001; significance relative to yeast: ^+^*P* ≤ 0.05, ^+++^*P* ≤ 0.001, and ^N.S^ *P* ≥ 0.05

## Discussion

Nanogold has recently demonstrated its ability to enter cancer cells and accumulate within them. Thus, NG has been shown to easily infiltrate the tumor vasculature and persist in tumors, resulting in their prolonged survival, which raised their concentration and ultimately resulted in tumor cell damage [30, 42]. It can also bind to other medications and chemicals, transporting them within tumor cells [43, 44]. It is non-toxic, and the kidneys can eliminate it [45]. Hendi et al. 2020 found that animals injected with NG (1–9 nm) had smaller tumors and a greater survival rate when compared to untreated control mice [30]. Many studies have demonstrated that NG may have tremendous potential for people suffering from metastases following breast cancer [46].

In this study, we used nanogold conjugated to heat-killed yeast for inducing apoptotic effect in tumor cells comparing this with the effect of heat-killed yeast with concentration (10^9^) alone in rats bearing breast cancer.

It was found that the balance of Bax/Bcl-2 heterodimers was critical in determining whether a cell survives or dies via programmed cell death. The mitochondrial mechanism of apoptosis may be modulated by anti-apoptotic and pro-apoptotic effectors, and it is commonly known that the preponderance of the pro-apoptotic protein (Bax) over the anti-apoptotic protein (Bcl-2) induces apoptosis. [47]. Our results showed a higher level of Bax and down-regulation of Bcl-2 in nanogold conjugated with heat-killed yeast than heat-killed yeast alone in comparison to the non-treated DMBA-induced tumor group. This coincides with the results of Ghoneum et al. (2008), and Elwakkad et al. (2018), who stated that heat-killed yeast induced apoptosis in the breast cancer cell line MDA 231 in vitro and in a skin tumor in vivo, respectively. This up-regulation of Bax and down-regulation of Bcl-2, with no negative effects on normal cells or other organs in animals, indicates that yeast promotes apoptosis via a mechanism involving intracellular Ca^2+^ and the Bax: Bcl-2 ratio [48].

The apoptosis is induced by the upregulation of Bax and the downregulation of Bcl2, disrupting the balance of the Bax/Bcl2 ratio, causing a change in the mitochondrial membrane potential and the release of cytochrome c from the mitochondrial membrane. Elwakkad et al. (2018) demonstrated that there was an increase in cytochrome c in heat-killed, treated skin cancer rats in vivo [22]. This result coincides with our results, which showed a higher increase in cytochrome c-treated rats with nanogold conjugated to heat-killed yeast than those treated with heat-killed yeast alone in comparison with non-treated tumors.

There was an increase in the levels of caspases 8, 3, and 9 that indicates the activation of the death receptor pathway for apoptosis, which can be explained by the increase of FasL in nanogold conjugated heat-killed yeast compared with rats treated by heat-killed yeast only in comparison with DMBA non-treated tumors. When the FasL homotrimeric protein binds to its receptor, it triggers oligomerization of the receptor. This is associated with the clustering of death domains and the binding of the cofactor FADD. The FADD protein interacts with a homologous domain in pro-caspase 8 via its DED (Death Effector Domain) motif. Upon recruitment by FADD, the complex of Fas, FADD, and pro-caspase 8 is known as the DISC (Death-inducing signaling complex); pro-caspase 8 oligomerization promotes its activation by self-cleavage. Active caspase 8 then activates downstream caspases 3 and 7 causing the cell to undergo apoptosis [49, 50].

When yeast conjugated to nanogold therapy was combined, it resulted in a drop in Bcl2 expression and an increase in Bax levels compared to rats with tumors alone. Furthermore, yeast conjugated to nanogold-treated rats had higher levels of cytochrome-c and FasL, as well as higher production of caspases 9, 8, and 3. This shows that treatment with yeast coupled to nanogold promotes apoptosis in breast cancer cells via both the mitochondrial and death receptor pathways.

Histopathological results showed changes reflecting the ability of nanogold-conjugated heat-killed yeast to induce apoptosis is greater than heat-killed yeast alone, as the nanogold conjugated with heat-killed yeast showed no tumor, no hyperplasia, no granulation tissue formation, no ulceration, and no suppuration. The heat-killed yeast showed residual atypical hyperplastic epithelial cells, no ulceration, no suppuration, and no granulation tissue formation.

Genetic investigation showed a significant increase in mRNA of Bcl2 in the tumor group to the normal (negative control) group and a very high increase in the yeast and nanogold-conjugated heat-killed yeast with no significant difference between the treated groups. There was a highly significant increase in gene expression of inflammatory cytokine TNF-α in positive control than in negative control, yeast, and nanogold conjugated heat-killed yeast. The gene expression of p53 showed depression in positive control in comparison to the negative control, yeast, and nanogold-conjugated heat-killed yeast.

Toxicological tests were conducted to establish whether yeast or nanogold-conjugated heat-killed yeast had any adverse effects on internal organs (liver and kidney) and the redox status of the animals.

DMBA causes oxidative stress by generating ROS and peroxides as a result of DMBA enzymatic activity [9] and this oxidative stress is important in carcinogenesis [10]. It enhances the amount of lipid peroxidation (LPO) in the rat-modeled group, which is implicated in carcinogenesis [51], explaining the depletion of GPX, SOD, and total antioxidants in the DMBA-treated animal’s group as compared to the normal control group. While there was a considerable increase in LPO in rats with breast cancer as compared to controls, antioxidants may protect against cancer by directly or indirectly scavenging free radicals and ROS. These findings corroborated our results, which revealed a reduction in GPX, SOD, and total antioxidant levels in the positive control group as compared to the negative control, nanogold conjugated heat-killed yeast, and heat-killed yeast groups [12, 52]. Our results showed an increase in LPO in the tumor group, which returned to normal with treatment with nanogold conjugated heat-killed yeast and with heat-killed yeast.

In this study, our results showed that the induction of breast cancer by DMBA resulted in a significant increase in the levels of ALT, AST, creatinine, and urea in animals when compared to the control group, nanogold conjugated heat-killed yeast and heat-killed yeast, because of DMBA’s harmful effects on hepatic cells and kidneys. This is coinciding with Hendi et al. 2020 and Chen et al. 2013 [30, 53]. It has been demonstrated that it caused hepatic damage [54, 55]. The treatment with nanogold conjugated to heat-killed yeast, heat-killed yeast caused the levels of ALT, AST, creatinine, and urea to decrease again to the normal ranges, and this was more observed in nanogold-conjugated to heat-killed yeast group indicating relatively healthy hepatic cells that agree with the findings of Jo et al. [56].

The combination between yeast and NG helps to increase their modulating effect against DMBA-induced breast cancer that is because NG influenced DMBA-induced breast cancer, reducing the incidence of tumors in the breast and lymph nodes; moreover, it enhanced biological processes such as liver and kidney functions that were disrupted by tumor presence [30].

There is growing evidence that NG possesses antioxidant capability [57], which decreases ROS formation while increasing antioxidative enzyme activities and decreasing pro-inflammatory molecule expression [58]. Thus, increases hepatic GPX, SOD activity, and total antioxidant capacity [59] that is corresponding to our findings.

The histopathological results of the liver and kidney showed normal tissue of nanogold-conjugated heat-killed yeast and heat-killed yeast in comparison to the negative control.

## Conclusion

Our results proved that the nanogold-conjugated heat-killed yeast can initiate apoptosis and gave better results as a treatment for breast cancer than heat-killed yeast alone. It is a novel method for in vivo cancer therapy as it can penetrate cancer cells and start their apoptosis without affecting the surrounding normal tissues. This, in turn, gives us new insight and future hope, for the first time, that breast cancer can be treated by a non-invasive, simple, safe, and naturally originated method and achieves a hopeful treatment for breast cancer without causing any harmful or undesirable side effects for patients.

## Data Availability

All data generated or analyzed during this study are included in this published article.

## Abbreviations

DMBA: 7,12-Dimethylbenz(a)anthracene
PGE2: Prostaglandin E2
PAHs: Polycyclic aromatic hydrocarbons
ROS: Reactive oxygen species
MBC: Metastatic breast cancer
*S.** cerevisiae*: *Saccharomyces cerevisiae*
GNPs: Gold nanoparticles
NG: Nanogold
*S. cerevisiae*–GNSs: *S. cerevisiae* Conjugated to gold nanospheres
IT: Intratumoral
UV-Vis: Ultraviolet-visible absorption spectra
TEM: Transmission electron microscope
ED: Electron diffraction
GNSs: Gold nanospheres
LSPR: Localized surface plasmon resonance
FWHM: Full width at half-maximum
PBS: Phosphate-buffered saline
H&E: Hematoxylin and eosin
qRT-PCR: Quantitative real-time polymerase chain reaction
TNF-α: Tumor necrosis factor-alpha
Bcl-2: B cell lymphoma 2
DCIS: Ductal carcinoma in situ
FADD: Death domains and binding of co-factor
DED: Death effector domain
DISC: Death inducing signaling complex
LPO: Lipid peroxidation
